# Transcriptome Analysis Reveals Potential Regulatory Genes Related to Heat Tolerance in Holstein Dairy Cattle

**DOI:** 10.3390/genes11010068

**Published:** 2020-01-07

**Authors:** Shenhe Liu, Tingting Yue, Muhammad Jamil Ahmad, Xiangwei Hu, Xinxin Zhang, Tingxian Deng, Yan Hu, Changjiu He, Yang Zhou, Liguo Yang

**Affiliations:** 1 Ministry of Education, Key Laboratory of Agricultural Animal Genetics, Breeding and Reproduction, College of Animal Science and Technology, Huazhong Agricultural University, Wuhan 430070, China; Liushenhe2015@163.com (S.L.); 13653809423@163.com (T.Y.); jameel_uaf@hotmail.com (M.J.A.); hu15827216812@163.com (X.H.); zxx1144795936@163.com (X.Z.); yanshen@webmail.hzau.edu.cn (Y.H.); chungjoe@mail.hzau.edu.cn (C.H.); yangzhou@mail.hzau.edu.cn (Y.Z.); 2 College of Animal Science and Veterinary Medicine, Henan Agricultural University, Zhengzhou 450046, China; 3 Guangxi Provincial Key Laboratory of Buffalo Genetics, Breeding and Reproduction Technology, Buffalo Research Institute, Chinese Academy of Agricultural Sciences, Nanning 530001, China; dtx282000@163.com

**Keywords:** heat tolerance, RNA-Seq, dairy cows, hub genes, milk yield

## Abstract

Heat stress affects the physiology and production performance of Chinese Holstein dairy cows. As such, the selection of heat tolerance in cows and elucidating its underlying mechanisms are vital to the dairy industry. This study aimed to investigate the heat tolerance associated genes and molecular mechanisms in Chinese Holstein dairy cows using a high-throughput sequencing approach and bioinformatics analysis. Heat-induced physiological indicators and milk yield changes were assessed to determine heat tolerance levels in Chinese Holstein dairy cows by Principal Component Analysis method following Membership Function Value Analysis. Results indicated that rectal temperature (RT), respiratory rate (RR), and decline in milk production were significantly lower (*p* < 0.05) in heat tolerant (HT) cows while plasma levels of heat shock protein (HSP: HSP70, HSP90), and cortisol were significantly higher (*p* < 0.05) when compared to non-heat tolerant (NHT) Chinese Holstein dairy cows. By applying RNA-Seq analysis, we identified 200 (81 down-regulated and 119 up-regulated) significantly (|log2fold change| ≥ 1.4 and *p* ≤ 0.05) differentially expressed genes (DEGs) in HT versus NHT Chinese Holstein dairy cows. In addition, 14 of which were involved in protein–protein interaction (PPI) network. Importantly, several hub genes (*OAS2*, *MX2*, *IFIT5* and *TGFB2*) were significantly enriched in immune effector process. These findings might be helpful to expedite the understanding for the mechanism of heat tolerance in Chinese Holstein dairy cows.

## 1. Introduction

Heat stress causes great economic loss to the livestock industry worldwide, particularly the dairy industry [1]. Increasing evidence suggests that heat stress not only affects the physiology, feed intake, and milk production but also the reproduction efficiency of dairy cattle [2,3,4]. Cumulatively, these responses cause annual economic losses between $ 897 and $ 1500 million to the dairy industry [5]. Modifications to combat heat stress in animals during summer include physical adjustment of environment, genetic improvement of heat tolerant breeds, and nutritional alterations [6]. A cost-effective way to alleviate heat stress is breeding for heat tolerance [7], and dairy cattle have variability in predisposition to heat tolerance [8]; hence the selection for heat tolerance and production can be exploited [9]. Moreover, Garner et al. [10] reported that selection for heat tolerance improves animal welfare and dairy farm productivity. This presumption highlights the importance of elucidating the regulation mechanism of heat tolerant in dairy cows.

Transcriptome sequencing has varied executions i.e., mRNA profiling and the compilation of non-coding RNA (ncRNA). RNA-Seq (RNA Sequencing) has been used primarily for transcriptome profiling that has the potential to create the list of expressed genes to sort out DEGs (differentially expressed genes) [11]. RNA-Seq is also capable to detect DEGs of complex traits such as milk production, reproduction, and meat quality [12,13,14]. However, inadequate knowledge of mRNA expressions profile for heat tolerant in Chinese Holstein dairy cows remains.

Therefore, this study aimed to characterize DEGs in blood samples for heat stress through RNA-Seq data analysis in heat tolerant (HT) and non-heat tolerant (NHT) high-yielding lactating Chinese Holstein dairy cows. This work may potentially be used in marker-assisted selection and development of high-yielding heat tolerant Chinese Holstein dairy cow lines.

## 2. Materials and Methods 

### 2.1. Animals and Housing

A total of 42 healthy, lactating Chinese Holstein dairy cows (age: 3–5 years) peak milk production (42 ± 0.90 kg/day), in post calving (105.28 ± 2.96 days) were used during the months of June and August 2018 in this study. In addition to peanut seedlings and green fodder, roughage and concentrate supplement were fed according to their body weight requirements [15], and clean water was available ad libitum. All the experimental designs and methods involving dairy cows were approved by the Huazhong Agricultural University Animal Care and Use Committee (HZAUCA-2018-006).

### 2.2. Experiment Design and Sample Collection

Heat stress indexes including physiological indicators and milk yield change were used to investigate the response of Chinese Holstein dairy cows (*n* = 42) to heat stress. First, rectal temperature (RT) and respiratory rate (RR) were detected and recorded at 1:00–3:00 pm for 7 consecutive days in June and August for each experimental cow according to previous methods [16]. Milk yield was recorded three times per day for 7 consecutive days at the beginning of June and August, respectively, and changes in milk yield per cow owing to heat stress was calculated as the difference in the milk yield between two months. Temperature-humidity index (THI), calculated by relative humidity (RH) and ambient temperature (AT), is an indicator of heat stress levels for animals and was measured according to [17], using the formula: THI = (1.8 × AT + 32) − (0.55 − 0.0055 × RH) × (1.8 × AT − 26), while the AT and RH were monitored for 12 h (7:00 am to 7:00 pm), once every two hours, for 7 consecutive days in June and August, respectively.

Aiming to screen HT and NHT dairy cows, we firstly performed principal component analysis (PCA) on the RT, RR and milk yield changes of 42 dairy cows. The contribution rate of first, second, and third component analysis were assessed and used for comprehensive evaluation of heat tolerance ability of cows. Then, the first and second principal components of 42 cows were subjected to membership function analysis to obtain the difference in heat tolerance of these cows, and the order of heat tolerance was evaluated by the magnitude of weighted membership function value, i.e., RW value. Finally, we selected the first three and last three dairy cows based on RW value, and named HT and NHT cows, respectively, for mRNA sequencing. A blood sample (10 mL) in duplicate for Chinese Holstein dairy cows (*n* = 6) was collected at 2:00 pm from the external juggler vein at another day in August (THI 86) and placed instantly in non-RNA-enzyme tube containing ethylene diamine tetraacetic acid (EDTA) and Trizol reagent (Invitrogen, Carlsbad, CA, USA), for plasma separation and RNA extraction, respectively. Plasma was extracted by centrifuging blood (3000 *g* for 15 min) to measure HSP70 (Mlbio, Shanghai, China), HSP90 (Mlbio), and cortisol (Mlbio) levels following ELISA instructions. All assays had intra- and inter-assay coefficients of variation of less than 10% and 15%, respectively. Studies have indicated that THI 68 is the threshold of heat stress for high milk yielding (>35 kg/day) lactating dairy cows [18]; hence we selected THI of 86 in August to identify the heat stressed dairy animals is feasible.

### 2.3. Transcriptome Profiling

For the RNA-Seq, Kit method was used for total RNA collection (Tiangen, Beijing, China) and purification (Illumina, San Diego, CA, USA) following the instructions provided by the manufacturer. The SDS-PAGE and Agilent Bio analyzer 2100 system (Agilent, Santa Clara, CA, USA) were used to assure the quality and quantity for total RNA, respectively. Each sample was run using the Illumina TruSeq™ RNA Sample Preparation Kit (Illumina) to create the cDNA library. 

Total RNA (5 μg) was used for the poly (A) mRNAs isolation using Oligo (dT) magnetic beads (Invitrogen). To create the final cDNA library, purification and amplification of cDNA was done using PCR, tracked by PCR enriched chemically fragmented ~ 200 nt fragments. The cDNA libraries were sequenced using Illumina HiSeq™ 2500 platform (Illumina). 

### 2.4. Analysis of RNA Sequencing Data

For the transcriptome data analysis, the raw data were quantified using the FastQC software to the default settings. The obtained clean reads were mapped to *Bos Taurus* genome (https://www.ncbi.nlm.nih.gov/genome/?term=bos+taurus) using HISAT2 ver. 2.1.0 [19]. We calculated the fragment Per Kilo bases per Million fragments (FPKM) values for the same gene set using RSEM [20]. The differential analysis was done using the DESeq2 R package [21], while the level of significance was (|log2fold change| ≥ 1.4 and *p*-value ≤ 0.05) for DEGs.

### 2.5. Characterization of SNP, Indel, and Alternative Splicing

Mapping (RNA-Seq) reads into the genome of *B. Taurus* was done using SAMtools [22] and GATK [23], the SNP and Indel analyses were performed. Alternative splicing (AS) events for each library were recorded using ASprofile [24].

### 2.6. Functional Annotation

Functional annotation regarding DEGs and hub genes was done by GO (Gene Ontology), functional analysis and the KEGG (Kyoto Encyclopedia of Genes and Genomes) pathways enrichment analysis using the KEGG Orthology-Based Annotation System (KOBAS) 3.0, setting the *p* ≤ 0.05 to identify their biological significance. 

The plot results were depicted using the ggplot2 [25] R-package. The STRING database (https://string-db.org/) was used to construct PPI (protein-protein interaction) network, and a confidence score > 0.4 and *p* < 0.05 was set as the cutoff criterion. 

### 2.7. Quantitative Real-Time PCR Confirmation

RNA-Seq results were validated using randomly selected seven genes by RT-qPCR. Primer 5.0 was used to design the Primers and sent to Sangon Biotech, Co. Ltd. (Shanghai, China) for synthesis (Table S1). The total RNAs were reverse transcribed into cDNA using RevertAid First Strand cDNA Synthesis Kit (ThermoFisher, Waltham, MA, USA) according to the instructions provided by the manufacturer. QuantiNova SYBR Green PCR Kit (QIAGEN, Shanghai, China) was used to run qPCR, whereas *GAPDH* gene was used to normalize the relative abundance of genes. All data from three technical replicates for each sample were analyzed using the 2-ΔΔCt method [26]. 

### 2.8. Statistical Analysis

All the data of physiology, milk yield, ELISA and qPCR were presented as mean ± standard error of the mean (M ± SEM). Student’s *t*-test was applied to determine the level of significance between the two samples. Differences were accepted as significant when adjusted *p* < 0.05 (Bonferroni). SAS 9.4 was used to execute PCA method (PRINCOMP Procedure) following Membership Function Value Analysis for screening HT and NHT individuals using calculation formulas:

(1) $$R\left(Xi\right)=\frac{Xi-X\min}{X\max-X\min}\;i=1,2,\ldots ,n$$

Indicating *Xi* is the value of *i*th principal component, *X*min and *X*max are the maximum and minimum values of *i*th principal component, respectively.

(2) $$Wi=Pi/\overset{n}{\underset{i=1}{\sum }}Pi\;i=1,2,\ldots ,n$$

where *Wi* is the weight of the *i*th principal component among all the principal components selected for evaluating heat tolerance in dairy cows, and *Pi* is the contribution rate of the *i*th principal component.

(3) $$\mathrm{RW}=\overset{n}{\underset{i=1}{\sum }}\left[R\left(xi\right)\times Wi\right]\;i=1,2,\ldots ,n$$

Indicating RW is the weighted membership value calculated with principal components for each cow under heat stress conditions for ranking the heat tolerance of cows.

## 3. Results

### 3.1. Animal Source Description

Results of PCA on the heat stress indexes of 42 cows showed that the contribution rates of the first, second, and third principal components were 71.22%, 26.90%, and 1.88% (Table S2), respectively, of which the first two principal components could contain 98.12% of the information on heat stress indexes. Results of membership function analysis of the first two principal components of 42 cows described the difference in heat tolerance of these cows, and the order of heat tolerance was evaluated by the magnitude of weighted membership function value, i.e., RW value (Table S3). Based on RW values, 150,183 (named HT183) was determined to possess the strongest heat tolerance, followed by 150,051 (named HT051) and 150,196 (named HT196). Similarly, 140,190, 130,712, and 150,065 (named NH190, NH712, and NH065, separately) were considered non-heat tolerance (Table S3). 

**Figure 1:**
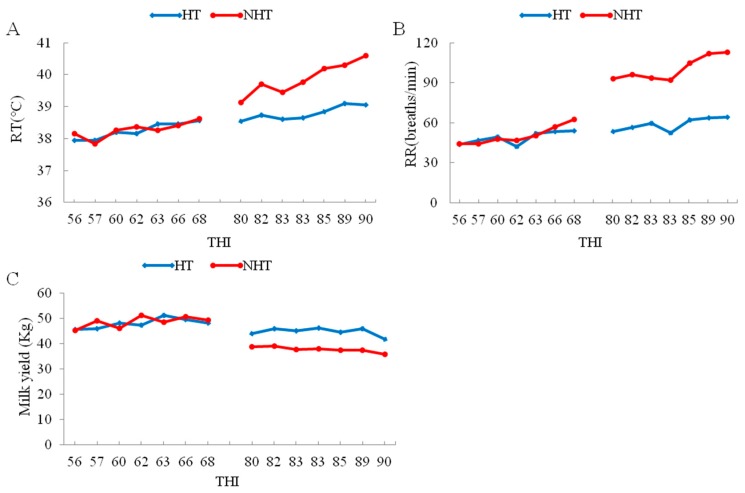
The change in respiratory rate (RR), rectal temperature (RT) and milk yield with increase of temperature-humidity index (THI) between HT and NHT Chinese Holstein dairy cows. (**A**) The change in RT with increase of THI; (**B**) The change in RR with increase of THI; (**C**) The change in milk yield with increase of THI. The left and right represent the changes in June and August, respectively.

Further, the data of heat stress indexes for the HT group (HT051, HT183, and HT196) and the NHT group (NH065, NH190, and NH712) cows shows that RT, RR and decline in milk yield were significantly lower (*p* < 0.05) for the HT vs. NHT group (Table 1, Figure 1), whereas the plasma levels for HSP70, HSP90 and cortisol were significantly higher (*p* < 0.05) in HT compared to NHT group (Table 1). Therefore, the selected two groups (HT and NHT) were feasible and used for further analysis.

### 3.2. Sequencing Data Summary

RNA-Seq analysis was performed to scan and characterize mRNA expression profiles linked to heat stress, in blood samples from HT (HT183 and HT196) and NHT (NH065, NH190, and NH712) animals while a sample (HT051) from the HT group was discarded because of degradation. On an average, the clean reads obtained were proximately 49.38 million and 54.19 million for HT (*n* = 2) and NHT (*n* = 3) cows, respectively, for further analysis (Table S2).

### 3.3. SNP, InDel and Alternative Splicing Analyses

We next executed the SNP, InDel and alternative splicing analysis for further study. The numbers of SNPs were detected 72,445, 71,666, 90,999, 55,911, and 85,797 in HT783, HT196, NH196, NHT065, and NHT712, respectively (Table S4). The number of InDel were found 5,139 and 4946 in HT183 and HT196, while 6451, 3975, and 6256 were identified in NH065, NH196, and NH712, respectively (Table S4). This screening process suggested that SNP variants were present in the reads aligned at the polymorphic locus from the mapped reference contigs, which was confirmed by total reads. High-confidence differences were composed of 90,999 SNPs and 6451 InDel involving more than one nucleotide. Statistical analyses suggested that, for both total and high-confidence SNPs, the proportion of transition nucleotide substitutions was greater than the proportion of transversions. Our results suggest that these SNPs can occur in both coding and non-coding regions of genes and may have functional consequences in terms of gene transcription or gene function. These functional consequences might be the biological cause of the association of SNPs with heat tolerance in dairy cow. Moreover, Skipped exon (SKIP 28,750 to 29,028), Multi-exon SKIP (MSKIP 8536 to 8604), Intron retention (IR 2842 to 3374), Multi-IR (MIR 350 to 426), Alternative exon ends (AE 9480 to 9625), Approximate SKIP (XSKIP 5008 to 5098), Approximate MSKIP (XMSKIP 1058 to 1,070), Approximate IR (XIR 1344 to 1590), Approximate MIR (XMIR 140 to 200), and Approximate AE (XAE 1021 to 1065) were identified in heat tolerant (HT) and non-heat tolerant (NHT). The top two AS events were SKIP and AE AS events, which accounted for more than 64.4% of AS events in each library (Table S4).

### 3.4. Differentially Expressed Gene Analyses

**Figure 2:**
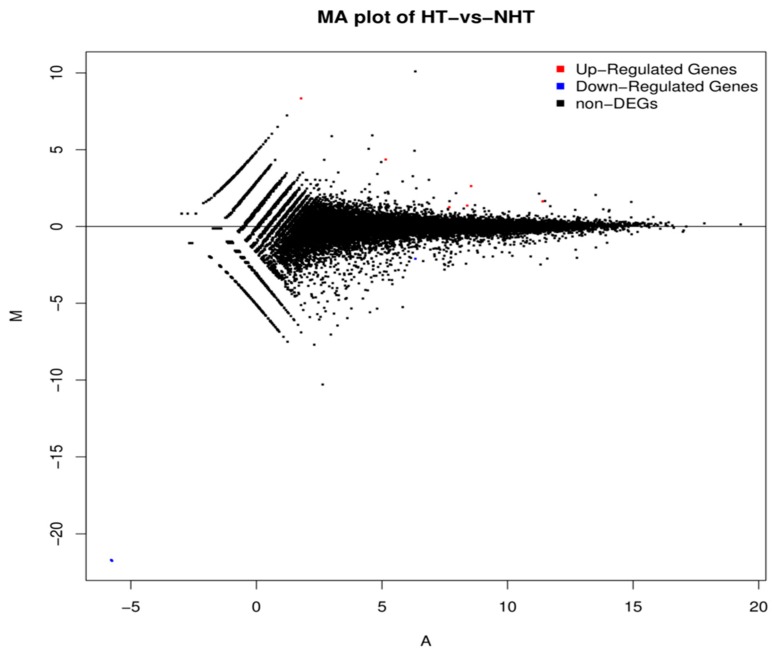
MA plot showing differentially expressed genes between HT and NHT Chinese Holstein dairy cows. M is the intensity ratio representing the *Y*-axis; A is the average intensity for a dot representing the *X*-axis. Red and blue dots represent up- and down-regulated genes, respectively while black dots represent the genes without significant differential expression.

**Figure 3:**
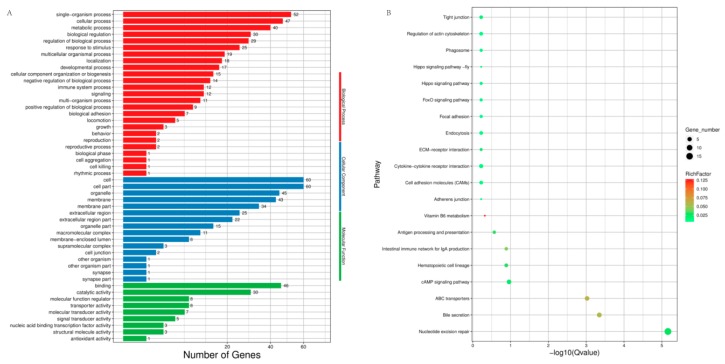
Functional annotation for the differentially expressed genes (DEGs). (**A**) Summary of Gene Ontology (GO) analysis present under three categories: molecular function, cellular component, and biological process; (**B**) The top 20 enriched Kyoto Encyclopedia of Genes and Genomes (KEGG) pathways for DEGs. Rich factor: the ratio of the number of genes divided by the number of all the genes in each pathway. Gene number: number of genes in each pathway.

The expression density distribution in five samples was consistent (Figure S1). The subsequent analysis identified a total of 200 DEGs in response to heat stress, including 81 down-regulated genes and 119 up-regulated genes in HT versus NHT (Figure 2, Table S5). The results of GO and KEGG analysis for the DEGs were listed in the Figure 3 (Table S6). The GO terms were enriched for biological process, cellular component, and molecular function. The DEGs significantly enriched in eight pathways, including Nucleotide excision repair (ko03420), Bile secretion (ko04976), ABC transporters (ko02010), cAMP signaling pathway (ko04024), Hematopoietic cell lineage (ko04640), Intestinal immune network for IgA production (ko04672), Antigen processing and presentation (ko04612), and Vitamin B6 metabolism (ko00750).

### 3.5. An Interaction Network of Differentially Expressed Genes

**Figure 4:**
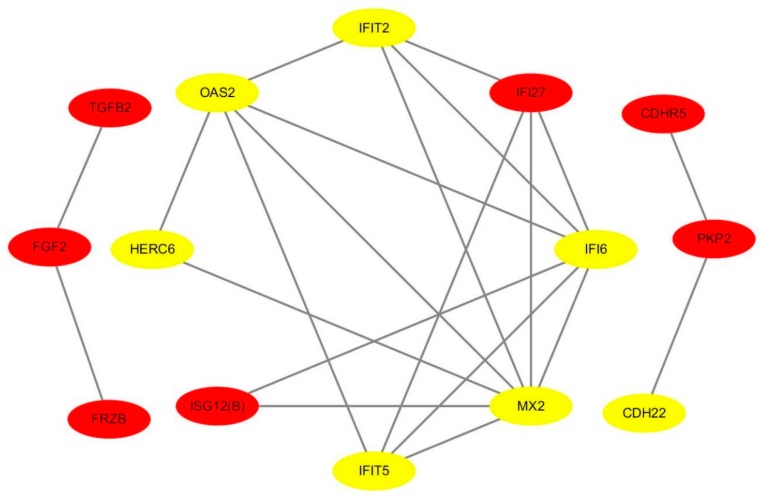
A protein–protein interaction (PPI) network of hub genes regarding heat stress. Yellow and red marker genes represent up- and down-regulated genes, respectively.

We identified 200 DEGs, 14 of which were within the PPI network diagram and treated as hub genes, including 7 up-regulated genes and 7 down-regulated genes (Figure 4).

Interestingly, 4 genes (*OAS2*, *MX2*, *IFIT5,* and *TGFB2*) were significantly enriched in the immune effector process (Top 1), suggesting that these genes might have a role in heat stress response (Table 2). 

**Figure 5:**
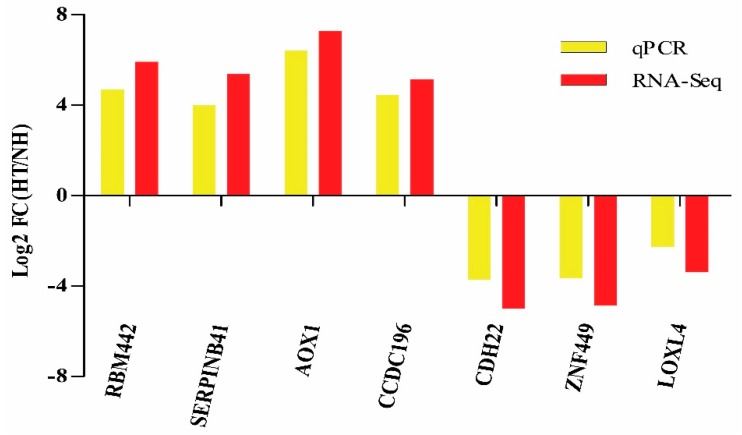
Validation of RNA-Seq results by qPCR. Bars of RNA-Seq and qPCR data were colored by black and white, respectively.

Moreover, the results of qPCR revealed that the expression level of 7 randomly selected genes coincides with that of the RNA-Seq (Figure 5).

## 4. Discussion

Heat stress has been of great concern for animal growth, development, and, in particular, the decline of reproductive efficiency in animals [27,28]. Combating against heat stress has always been a keen interest of farmers and researchers to reduce losses in livestock. There is a need for the measurement of a heat stress index for deeper insights into underlying biological pathways of heat stress and the productive performance in livestock. Nonetheless, physiological indicators (RT, RR) of heat tolerance in cattle can measure the heat stress induced changes in homeostasis [29]. Verma et al. [30] determined RR and RT as the most sensitive indices of heat tolerance among all the physiological reactions studied. This study showed that the RT and RR in HT individuals were significantly lower when compared to NHT, similar to observation in cattle [31] and buffalo [32]. Elevated temperature increased the plasma HSP90 and HSP70 levels more markedly in HT than in NHT individuals, coinciding to previous study in cattle [33] and rodents [34]. Higher plasma cortisol levels observed in the HT than in NHT individuals after the heat stress challenge in the present study is consistent with those reported by Hammond, et al. [35] in heat tolerant Romosinuano (RO) and heat-sensitive Angus (ANG) heifers after heat stress, and Shenhe et al. [16] in Mediterranean and Crossbred buffalo after heat stress. Thus, our findings showed that the selection of HT and NHT Chinese Holstein dairy cows is practical and can be used as reference for further analysis.

Transcriptome sequencing has the potential to recognize the candidate genes for the complex traits or disease. Several studies in cattle [36], poultry [37], and swine [38], highlighted the genes of interest for heat stress in animals. Nonetheless, it is difficult to understand the multifaceted changes at molecular level in response to heat stress in the animals. To date, little is known about the identification of candidate genes related to heat tolerance in Chinese Holstein dairy cows. 

We reported a total of 200 DEGs including 81 down regulated and 119 up regulated. The KEGG enrichment analysis revealed that nucleotide excision repair mechanism, bile secretion pathways, the ATP-binding cassette transporters, cAMP signaling pathway, and hematopoietic cell lineage were related to heat stress. Previously heat stress (43 °C) reported to reduce the nucleotide excision repair of DNA lesions caused by UV-B in fibroblast and keratinocytes [39], and the increased UV resistance was likely mediated through nucleotide excision repair [40]. ABC transporters may function in stress response [41]. The cAMP signaling pathway has a role in adaptation to heat stress and survival during host infection [42]. 

Identification of hub genes is crucial to elucidate the heat-resistant mechanisms. A total of 14 among 200 identified DEGs were within the PPI network, and involved in multiple GO terms and pathways related to heat stress. Nonetheless, the results of GO analysis indicated that several genes (*OAS2*, *MX2*, *IFIT5* and *TGFB2*) were most significantly enriched in immune effector process. Collier, et al. [43] have reported that the immune response system was activated in response to heat stress mediated by a series of cascades; HSF1 activation leads to an increased expression of heat shock proteins, reduction in fatty acid metabolism and resulted in endocrine system activation of the stress response. Moreover, our outcomes revealed that *TGFB2* and *FGF2* are part of MAPK signaling pathway. Previous work has discussed the activation of MAPK signaling pathway mediated by several extra-cellular stimuli, including UV radiation, osmotic shock and heat shock [32,36,44,45]. Of note, *TGFB2* as stress-related gene was significantly induced when human lung fibroblast cell lines were exposed to radiofrequency radiation [46]. 

Meanwhile, numerous studies coincide that lists of DEGs in our findings were associated to heat stress. For example, *ALAS2* has a role in protection against oxidative stress [47], *AOX1* transcript and protein abundance increased sharply under heat stress [48]. Heat stress also enhanced *AQP1* expression in cell [49]. Chinese medicine decoction supplementation significantly reduced the negative effects of heat stress on pig jejunum, partly owing to increased *SCT* expression [50]. The expression of *BPI* in fish was significantly up-regulated post crowding stress exposure [51]. Heat stress increased the expression of *GPX2* (2.23-fold change) relative to thermo-neutral conditions in rat ileum [52]. Heat stress improved the *EGF* mRNA expression, and the production of EGF or EGF-like peptides under heat stress may be an important survival mechanism in mouse mammary epithelial cells [53]. Heat stress also enhanced the expression levels of *IFIT1* and *IFIT2*, which can be bound to the translation initiation factor eIF3, inhibit translation of cellular stress response [54]. Thus, the capacity of heat tolerant altered expression of these gene groups could potentially be associated with the physiological and milk trait differences observed in Chinese Holstein dairy cows.

We designed this study with a limited number of animals to obtain deeper insight and find diversity between HT and NHT groups. A limited number of animals may limit the power of design as well as validity of results. However, several studies published previously using even the fewer animals compared with our study [32,55]. The results of our study are giving foundation for heat tolerance mechanism in dairy cows. We identified 200 DEGs in this study of which several genes (including *IGFB2*, *OAS2*, *MX2*, *IFIT5*, *FGF2*, *ALAS2*, *AOX1*, *AQP1*, *SCT*, *TGFB2*, *BPI*, *GPX2*, *EGF*, and *IFIT2*) were associated with heat tolerance and can use for Marker Assisted selection programmer to improve heat tolerance and minimize production loss in dairy cows. Future recommendations to further explore heat tolerance mechanism in dairy cows are to repeat the experiment globally in different climates with different breeds and a bigger number of experimental animals.

## 5. Conclusions

We compared the heat stress indexes between HT and NHT dairy cows, indicating that HT dairy cows had a significantly lower RR, RT and decline in milk yield and displayed a higher plasma cortisol and heat shock protein (HSP90 and HSP70) levels compared to NHT dairy cows. A total of 200 DEGs were identified between HT and NHT dairy cows. Importantly, four genes (*OAS2*, *MX2*, *IFIT5*, and *TGFB2*) related to heat tolerance were identified, which is involved in the immune effector process. These findings will help in exploring underlying heat-tolerant mechanisms in dairy cows.

## Figures and Tables

**Table 1:** Comparison of heat stress indexes between heat tolerant (HT) and non-heat tolerant (NHT) Chinese Holstein dairy cows (M ± SEM).

	HT (*n* = 3)	NHT (*n* = 3)
HSP70 (pg/mL)	391 ± 59 ^a^	183 ± 12 ^b^
HSP90 (pg/mL)	2747 ± 196 ^a^	1075 ± 84 ^b^
Cortisol (ng/mL)	247 ± 19 ^a^	144 ± 11 ^b^
RT (°C)	38.7 ± 0.02 ^a^	39.7 ± 0.05 ^b^
RR (breaths/min)	57 ± 1 ^a^	100 ± 6 ^b^
Decline in milk yield (kg)	2.8 ± 0.9 ^a^	10.7 ± 2.4 ^b^

RT = Rectal Temperature; RR = Respiratory Rate; ^a,b^ different superscripts differ significantly at *p* < 0.05.

**Table 2:** GO terms and KEGG enrichment pathways (Top 3) for hub genes.

Category	Term	ID	*p*-Value	Gene Symbol
KEGG	Proteoglycans in cancer	bta05205	0.004893	*FGF2*, *TGFB2*
	MAPK signaling pathway	bta04010	0.007510	*FGF2*, *TGFB2*
	Pathways in cancer	bta05200	0.017469	*FGF2*, *TGFB2*
GO	Immune effector process	GO:0002252	0.000020	*OAS2*, *MX2*, *IFIT5*, *TGFB2*
	Negative regulation of cartilage development	GO:0061037	0.000033	*FRZB*, *TGFB2*
	Defense response to virus	GO:0051607	0.000056	*OAS2*, *MX2*, *IFIT5*

## Supplementary Materials

The following are available online at https://www.mdpi.com/2073-4425/11/1/68/s1, Figure S1: The expression density distribution in five samples, Table S1: Primers for DEGs in RNA-seq, Table S2: Eigen values and variance contribution ratio of measured indicators and characteristic value of principal components, Table S3: Membership value of 42 dairy cows, Table S4: Description of the RNA-Seq data in the 5 dairy cow blood tissues, Table S5: Description of the DEGs, Table S6: The GO and KEGG analysis of DEGs.
