# Supplementary figures and images for: Transcriptome Analysis Reveals Potential Regulatory Genes Related to Heat Tolerance in Holstein Dairy Cattle
Source: Genes (Basel). 2020 Jan 7;11(1):68. doi: 10.3390/genes11010068 (PMC7017222; doi:10.3390/genes11010068)

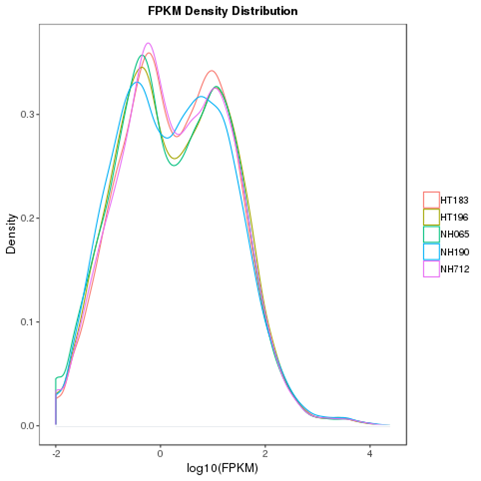

Supplement: Supplementary file 1 [file genes-11-00068-s001.zip › Supplementary File for Genes/S1 Figure.png]
